# Population-Based Study of Docetaxel or Abiraterone Effectiveness and Predictive Markers of Progression Free Survival in Metastatic Castration-Sensitive Prostate Cancer

**DOI:** 10.3389/fonc.2021.658331

**Published:** 2021-05-07

**Authors:** Juan Briones, Maira Khan, Amanjot K. Sidhu, Liying Zhang, Martin Smoragiewicz, Urban Emmenegger

**Affiliations:** ^1^ Division of Medical Oncology, Odette Cancer Centre, Sunnybrook Health Sciences Centre, Toronto, ON, Canada; ^2^ Department of Medicine, University of Toronto, Toronto, ON, Canada; ^3^ Biological Sciences Research Platform, Sunnybrook Research Institute, Sunnybrook Health Sciences Centre, University of Toronto, Toronto, ON, Canada; ^4^ Institute of Medical Science, University of Toronto, Toronto, ON, Canada

**Keywords:** metastatic castration sensitive prostate cancer (mCSPC), docetaxel (DOC), abiraterone (AA), retrospective analysis, real-world effectiveness

## Abstract

**Background:**

Both Docetaxel (DOC) and Abiraterone (ABI) improve the survival of men with metastatic, castration sensitive prostate cancer (mCSPC). However, the outcome among mCSPC patients is highly variable, while there is a lack of predictive markers of therapeutic benefit. Furthermore, there is limited data on the comparative real-world effectiveness of adding DOC or ABI to androgen deprivation therapy (ADT).

**Methods:**

We conducted a retrospective analysis of 121 mCSPC patients treated at Odette Cancer Centre (Toronto, ON, Canada) between Dec 2014 and Mar 2021 (DOC n = 79, ABI n = 42). The primary endpoint studied was progression free survival (PFS), defined as the interval from start of ADT to either (i) biochemical, radiological, or symptomatic progression, (ii) start of first-line systemic therapy for castration-resistant prostate cancer (CRPC), or (iii) death, whichever occurred first. To identify independent predictive factors for PFS in the entire cohort, a Cox proportional hazard model (stepwise selection) was applied. Overall survival (OS) was among secondary endpoints.

**Results:**

After a median follow-up of 39.6 and 25.1 months in the DOC and ABI cohorts, respectively, 79.7% of men in the DOC and 40.5% in the ABI group experienced a progression event. PFS favored the ABI cohort (p = 0.0038, log-rank test), with 78.0% (95%CI 66.4–91.8%) of ABI *versus* 67.1% (57.5–78.3%) of DOC patients being free of progression at 12 months. In univariate analysis superior PFS was significantly related to older age at diagnosis of mCSPC, metachronous metastatic presentation, low-volume (CHAARTED), and low-risk (LATITUDE) disease, ≥90% PSA decrease at 3 months (PSA90), and PSA nadir ≤0.2 at 6 months. Age (HR = 0.955), PSA90 (HR = 0.462), and LATITUDE risk stratification (HR = 1.965) remained significantly associated with PFS in multivariable analysis. OS at 12 months was 98.7% (96.3–100%) and 92.7% (85.0–100%) in the DOC and ABI groups (p = 0.97), respectively.

**Conclusions:**

In this real-world group of men undergoing treatment intensification with DOC or ABI for mCSPC, we did not find a significant difference in OS, but PFS was favoring ABI. Age at diagnosis of mCSPC, PSA90 at 3 months and LATITUDE risk classification are predictive factors of PFS in men with mCSPC.

## Introduction

In developing and developed countries, the age-adjusted incidence of prostate cancer has risen with time (1). Currently, between 4 and 8% of North American men diagnosed with prostate cancer present with *de novo* metastatic disease (2), and approximately 10–15% develop distant metastases after local therapy with curative intent (3). Hence, there is a significant number of prostate cancer patients who ultimately will die of metastatic disease.

The contemporary standard of care for metastatic castration-sensitive prostate cancer (mCSPC) comprises androgen deprivation therapy (ADT) combined with either second generation androgen receptor signaling inhibitors (ARSIs) such as Abiraterone (ABI) (4, 5), Enzalutamide (6, 7) or Apalutamide (8), or with Docetaxel (DOC) chemotherapy (9–11). Moreover, local radiotherapy prolongs overall survival (OS) in low volume mCSPC (12, 13). Numerically, all of these interventions improved median overall survival (OS) in a clinically meaningful way compared to ADT alone with hazard ratios (HRs) between 0.61 and 0.88 (14). With the availability of such a broad therapeutic arsenal consisting of agents with different toxicity profiles, clinicians can choose the most suitable treatment option depending on associated comorbidities and burden as well as distribution of disease (15, 16).

Nonetheless, the rapid therapeutic advances over the last few years have also created challenges. First of all, there is no definite data on the comparative efficacy of the varied treatment modalities. While an opportunistic comparison of men recruited simultaneously for the ADT + ABI and ADT + DOC arms of the STAMPEDE trial (arms G and C, respectively) suggests a similar OS benefit, ARSIs appear to be the preferred treatment option in meta-analyses compared to DOC when applying surface under the cumulative ranking analysis (17–19). On the other hand, it is worth noting that DOC is more cost-effective than ABI (20–22). Secondly, there are no validated predictive (bio)markers of response to ARSIs or DOC (23). Thirdly, the clinical studies differed with respect to the eligibility criteria used, resulting in study cohorts with varying risk profiles, be it related to high *versus* low volume disease as defined in the CHAARTED study, high risk disease as outlined in the LATITUDE trial, or recurrent *versus de novo* mCSPC (4, 9, 24). As for the CHAARTED high volume and LATITUDE high risk definitions, they are in excellent but not perfect agreement, and both predict worse outcome (25–27). Finally, numerous prostate-specific antigen (PSA) based response parameters are associated with oncological outcome [*e.g.*, absolute PSA nadir of ≤0.2 ng/ml, ≥50 or ≥90% PSA response (PSA50 and PSA90, respectively), and median time to PSA nadir], but they are not helpful for the initial treatment decision between DOC and ARSIs (28–30).

Based on the above, we decided to study the performance of ABI and DOC under real-world conditions when added to ADT for the treatment of mCSPC to compare the oncological activity of these drugs and to identify predictive markers of response.

## Patients and Methods

### Patient Selection and Treatment Details

This retrospective study collected data from 121 mCSPC patients treated at Odette Cancer Centre (Toronto, ON, Canada) between December 2014 and March 2021. Patient and treatment characteristics were extracted from SunnyCare (in-house electronic health information system). Eligible patients had mCSPC based on computed tomography (CT), bone scan, or both, and had to be on treatment with ADT with documented castrate testosterone (≤1.7 nmol/L).

DOC or ABI were prescribed at the discretion of the treating oncologist. DOC became provincially available for mCSPC in the second half of 2014, whereas ABI was approved by Health Canada in February 2018 and was made available *via* access program shortly thereafter for men with *de novo* high-risk mCSPC as per LATITUDE criteria.

The DOC regimen consisted of 75 mg/m^2^, initiated without prednisone, every 3 weeks for a maximum of six cycles. Patients were clinically assessed before each cycle of chemotherapy. Similarly, hematologic, hepatic, and renal functions as well as PSA were measured at baseline and before each cycle of chemotherapy. Thereafter, patients were usually evaluated every 3 months until progression of disease. Aside from baseline imaging, patients underwent repeat imaging at completion of DOC and every 6–12 months thereafter, or earlier as clinically indicated.

ABI was given at a dose of 1,000 mg once daily (without food), combined with prednisone 5 mg once daily, and continued until either progression of disease or intolerance, whichever occurred first. Mostly, patients were assessed monthly for the first three months, and three-monthly thereafter. Routine bloodwork (including liver profile, creatinine, electrolytes, and PSA) was obtained biweekly for the first three months, and then every three months. Bone and CT scans were done at the treating physician’s discretion, typically ≤12 months after start of ABI and six-monthly thereafter.

All study activities were approved by the Research Ethics Board of Sunnybrook Research Institute (Toronto, ON, Canada).

### Endpoints

The predefined primary endpoint was progression free survival (PFS), defined as the interval from start of ADT to either (i) biochemical (applying the PCWG3 criteria (31)), radiological, or symptomatic progression, (ii) start of first-line systemic therapy for castration-resistant prostate cancer (CRPC), or (iii) death, whichever occurred first. Patients without progression event were censored at the last follow-up date or the data cutoff on March 15, 2021. Key secondary endpoints were OS, time to castration resistant prostate cancer (CRPC), and time to start of first line of systemic therapy for CRPC, all of those calculated from the start of ADT to the event of interest. PSA dynamics were also assessed as secondary endpoints, including the PSA nadir ≤0.2 ng/mL rate at 6 months as well as PSA50 and PSA90 response rates at 3 and 6 months.

### Statistical Analysis

Patient and treatment characteristics of the DOC and ABI cohorts were compared using the Wilcoxon rank-sum non-parametric test for continuous variables or the Fisher exact test for categorical variables. PFS, OS, time to CRPC, and time to start of first line systemic therapy for CRPC were estimated by the Kaplan–Meier method, with log-rank test to compare the DOC *versus* ABI cohorts.

To search for predictive factors of PFS in the total population (n = 121), we applied univariate and multivariable (*i.e.*, backward stepwise selection procedure) Cox proportional hazard models, the latter adjusted for age and the Charlson Comorbidity Index (CCI). Hazard ratios (HRs) and 95% confidence intervals (CIs) were calculated for each covariate. Natural log transformation was used for some variables to normalize the distribution. The generalized R^2^ was calculated based on the likelihood ratio statistic (LRT) for testing the global null hypothesis (32), using the formula of

$$R^{2}\;=1-e^{-(LRT/n)},$$

where LRT = −2logL(0) − [−2logL(p)], n = sample size used, logL(0) = log-likelihood for a null model with no covariates, and logL(p) = log-likelihood for the fitted model with p covariates. R^2^ (between 0 and 1) is larger when the covariates are more strongly associated with the dependent variable.

Two-sided p-values <0.05 were considered statistically significant. Missing data was handled as real missing values, *i.e.*, the complete data set was used since missing data was marginal (<5%). All analyses were conducted using Statistical Analysis Software (SAS version 9.4, Cary, NC) and R package (v3.6.1).

## Results

### Patient and Treatment Characteristics

A total of 121 patients met the eligibility criteria, of which 79 were treated with DOC and 42 received ABI. Patient characteristics are detailed in **Table 1**. While the patient cohorts were comparable overall, DOC patients were younger [mean age ± standard deviation (SD) 65.76 ± 8.25 *versus* 72.40 ± 7.92 years] and had a lower CCI (mean ± SD 8.52 ± 1.10 *versus* 9.55 ± 1.58).

**Table 1:** Patient and treatment characteristics.

	Abiraterone(N = 42)	Docetaxel(N = 79)	p-value
***Demographics***			
**Age at diagnosis of mCSPC (years)**			**<.0001**
Mean ± SD	72.40 ± 7.92	65.76 ± 8.25	
Median (Inter-quartiles)	73.5 (68.0, 79.0)	66.0 (60.0, 71.0)	
Min, Max	50.0, 85.0	44.0, 90.0	
Initial stage			0.5536
Localized	17 (40.48%)	27 (34.18%)	
Metastatic	25 (59.52%)	52 (65.82%)	
Gleason score			0.7240
6	3 (7.89%)	4 (5.56%)	
7	6 (15.79%)	15 (20.83%)	
8–10	29 (76.32%)	53 (73.61%)	
Local treatment			0.4226
No	25 (59.52%)	54 (68.35%)	
Yes	17 (40.48%)	25 (31.65%)	
Prior neo/adjuvant ADT			0.6212
No	33 (78.57%)	66 (83.54%)	
Yes	9 (21.43%)	13 (16.46%)	
Bone metastases			0.2554
No	3 (7.14%)	12 (15.19%)	
Yes	39 (92.86%)	67 (84.81%)	
Lymph nodes metastases			0.9597
No	20 (47.62%)	38 (48.10%)	
Yes	22 (52.38%)	41 (51.90%)	
Visceral metastases			0.6493
No	32 (76.19%)	63 (79.75%)	
Yes	10 (23.81%)	16 (20.25%)	
CHAARTED criteria			0.0866
Low volume	7 (16.67%)	25 (31.65%)	
High volume	35 (83.33%)	54 (68.35%)	
LATITUDE criteria			0.1420
Low risk	9 (23.68%)	30 (39.47%)	
High risk	29 (76.32%)	46 (60.53%)	
**Charlson Comorbidity Index**			**<.0001**
Mean ± SD	9.55 ± 1.58	8.52 ± 1.10	
Median (Inter-quartiles)	9.0 (9.0, 10.0)	9.0 (8.0, 9.0)	
Min, Max	7.0, 16.0	6.0, 13.0	
PSA at start of ADT for mCSPC			0.4791
Mean ± SD	308.455 ± 797.506	280.985 ± 718.502	
Median (Inter-quartiles)	33.73 (7.24, 107.00)	32.63 (11.68, 231.00)	
Min, Max	1.78, 3761.00	0.13, 5000.00	
***Treatment information***			
No. of cycles of DOC			NA
1-5	NA	5 (6.33%)	
6		74 (93.67%)	
Days from start of ADT to start of ABI/DOC			0.7604
Mean ± SD	63.12 ± 39.47	66.32 ± 62.12	
Median (Inter-quartiles)	52.0 (35.0, 98.0)	48.0 (37.0, 71.0)	
Min, Max	2.0, 162.0	18.0, 437.0	
**Reason for treatment discontinuation**			**0.0119**
Disease progression	6 (14.29%)	1 (1.27%)	
Toxicity/adverse event	2 (4.76%)	4 (5.06%)	
Not applicable	34 (80.95%)	74 (93.67%)	

ABI, abiraterone; ADT, androgen deprivation therapy; DOC, docetaxel; mCSPC, metastatic castration-sensitive prostate cancer; PSA, prostate specific antigen.

The median time from the start of ADT to initiation of either DOC or ABI was similar (48 days [interquartile range (IQR) 37,71] *versus* 52 days (35,98); p = 0.76). The majority of chemotherapy patients completed six cycles of DOC (74/79, 93.67%). Side effects accounted for DOC or ABI discontinuation in around 5% of patients each.

### PSA-Based Outcome Parameters

The median PSA values at start of ADT were similar between both cohorts, 32.63 ng/ml (IQR 11.68,231.00) in patients treated with DOC and 33.73 ng/ml (7.24,107.00) in the ABI cohort (**Table 1**). While the majority of patients in both cohorts achieved a PSA decrease to well below 1 ng/ml, PSA nadir-based parameters favored ABI patients. On the other hand, there were no statistically significant differences between the two groups in terms of PSA50 and PSA90 response rates (**Table 2**).

**Table 2:** PSA-based outcome parameters.

	Abiraterone(N = 42)	Docetaxel(N = 79)	p-value
**PSA nadir**			**0.0007**
N	26	75	
Mean ± SD	0.469 ± 1.801	2.108 ± 4.609	
Median (Inter-quartiles)	0.02 (0.02, 0.09)	0.20 (0.02, 1.35)	
Min, Max	0.02, 9.20	0.02, 24.59	
**PSA nadir categories**			**0.0061**
<0.2	22 (84.62%)	37 (49.33%)	
0.2–4.0	3 (11.54%)	26 (34.67%)	
>4.0	1 (3.85%)	12 (16.00%)	
**PSA nadir ≤ 0.2 *at 6 months***			**0.0155**
No	15 (57.69%)	62 (82.67%)	
Yes	11 (42.31%)	13 (17.33%)	
Days from start of ADT to PSA nadir			0.5733
Mean ± SD	232.88 ± 152.05	225.91 ± 104.73	
Median (Inter-quartiles)	172.5 (128.0, 338.0)	204.0 (154.0, 299.0)	
Min, Max	83.0, 736.0	35.0, 497.0	
PSA at 3 months from start of ADT			0.1985
N	41	77	
Mean ± SD	14.784 ± 46.321	6.963 ± 13.217	
Median (Inter-quartiles)	0.27 (0.10, 3.03)	0.91 (0.20, 6.59)	
Min, Max	0.02, 259.70	0.02, 61.98	
PSA at 6 months from start of ADT			0.3240
N	39	73	
Mean ± SD	6.492 ± 17.505	3.867 ± 9.826	
Median (Inter-quartiles)	0.20 (0.03, 1.00)	0.30 (0.05, 2.49)	
Min, Max	0.02, 74.93	0.02, 69.69	
PSA50 at 3 months from start of ADT			0.3400
No	3 (7.32%)	2 (2.60%)	
Yes	38 (92.68%)	75 (97.40%)	
PSA90 at 3 months from start of ADT			0.6339
No	7 (17.07%)	17 (22.08%)	
Yes	34 (82.93%)	60 (77.92%)	
PSA50 at 6 months from start of ADT			0.2774
No	2 (5.13%)	1 (1.37%)	
Yes	37 (94.87%)	72 (98.63%)	
PSA90 at 6 months from start of ADT			0.4419
No	5 (12.82%)	14 (19.18%)	
Yes	34 (87.18%)	59 (80.82%)	

ADT, androgen deprivation therapy; PSA, prostate specific antigen.

### Progression Free Survival

**Figure 1:**
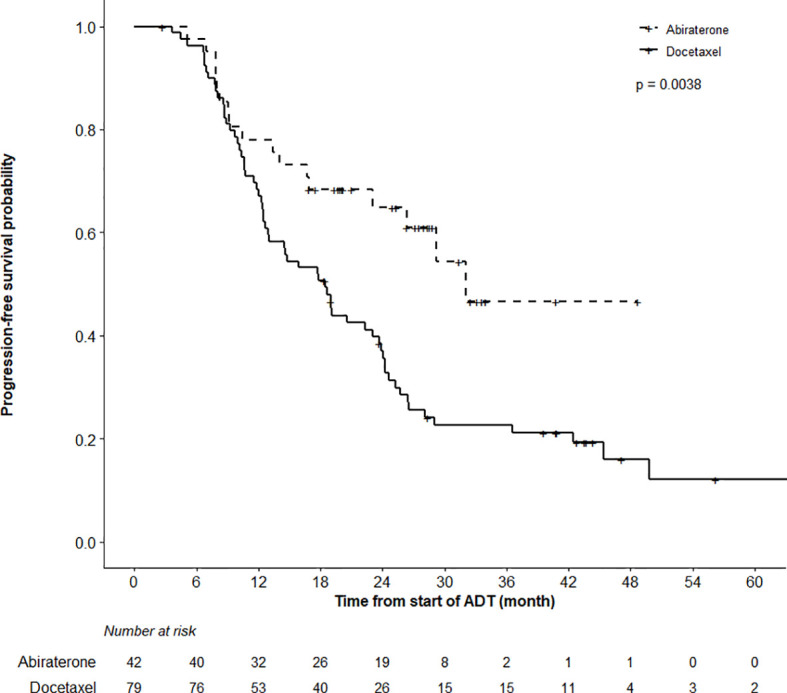
Progression Free Survival (PFS) Analysis. PFS analysis in patients undergoing abiraterone versus docetaxel therapy until month 48 revealed superior outcome in abiraterone patients (log-rank test, p = 0.0038).

After a median follow-up of 39.6 (IQR 26.9,47.2) and 25.1 (IQR 17.5, 30.9) months in the DOC and ABI cohorts, respectively, 79.7% of men in the DOC and 40.5% in the ABI group experienced a progression event (**Figure 1**). PFS was triggered by biochemical progression in 71% of patients in the DOC group and in 80% of men in the ABI group. The remaining events were triggered by radiological changes. The actuarial median PFS was 18.5 months (95%CI 12.6–23.7) in the DOC cohort, and 32.0 months (95%CI 23.1–48.7) in the ABI cohort. While neither the median PSA nor the median radiological PFS was reached in men undergoing ABI therapy, the according medians were 24.2 and 26.8 months in the DOC group. Overall, the PFS analysis favored ABI (p = 0.0038).

### Predictive Factors of Progression Free Survival

Univariate analysis of the entire study cohort (**Table 3**) identified six factors significantly related to PFS. Older age at diagnosis of mCSPC (HR = 0.969), PSA90 at 3 months (HR = 0.472), and PSA nadir ≤0.2 at 6 months (HR = 0.524) translated to better outcome, whereas PFS was shorter in men with high-risk disease features according to the LATITUDE criteria (HR = 1.653), high-volume disease as per CHAARTED criteria (HR = 1.722), and *de novo* metastatic disease (HR = 1.737). To account for significant differences regarding age and comorbidities in the ABI *versus* DOC cohorts (**Table 1**), the final multivariable model (**Table 4**) was adjusted for age and CCI. It revealed three factors that remained significant: age at diagnosis of mCSPC (HR = 0.955), PSA90 at 3 months (HR = 0.462), and the LATITUDE risk classification (HR = 1.965).

**Table 3:** Predictive factors of progression-free survival: univariate analysis.

Predictive factors	P-value	HR	95% CI of HR		R^2^ (%)
**Age at diagnosis of mCSPC (years)**	**0.0246**	**0.969**	**0.942**	**0.996**	**4.06**
**Initial stage (metastatic vs. localized)**	**0.0256**	**1.737**	**1.070**	**2.822**	**4.32**
Gleason score ≥8 (yes *vs*. no)	0.1843	1.477	0.831	2.625	1.71
Visceral metastasis (yes *vs*. no)	0.5068	0.826	0.470	1.452	0.38
**CHAARTED criteria (high *vs*. low volume)**	**0.0439**	**1.722**	**1.015**	**2.923**	**3.61**
**LATITUDE criteria (high *vs.* low risk)**	**0.0496**	**1.653**	**1.001**	**2.729**	**3.53**
Charlson Comorbidity Index ≥9 (yes *vs*. no)	0.6846	0.912	0.586	1.421	0.14
Days from start of ADT to start of ABI or DOC (log)	0.7822	1.046	0.759	1.443	0.06
PSA at start of ADT (log)	0.3670	1.049	0.946	1.164	0.67
PSA50 at 3 months from start of ADT (yes *vs*. no)	0.2914	0.579	0.209	1.599	0.81
**PSA90 at 3 months from start of ADT (yes *vs*. no)**	**0.0037**	**0.472**	**0.285**	**0.784**	**6.13**
**PSA nadir ≤0.2 at 6 months (yes *vs*. no)**	**0.0437**	**0.524**	**0.280**	**0.982**	**4.50**

ABI, abiraterone; ADT, androgen deprivation therapy; DOC, docetaxel; mCSPC, metastatic castration-sensitive prostate cancer; PSA, prostate specific antigen.

**Table 4:** Predictive factors of progression-free survival: multivariable analysis, adjusted for age and Charlson Comorbidity Index.

Multivariable Model	P-value	HR	95% CI of HR		R^2^ (%)
**Age at diagnosis of mCSPC (years)**	**0.0253**	**0.955**	**0.918**	**0.994**	**15.80**
Charlson Comorbidity Index ≥9 (yes *vs*. no)	0.1066	1.679	0.895	3.150	
**LATITUDE criteria (high *vs*. low risk)**	**0.0116**	**1.965**	**1.163**	**3.320**	
**PSA90 at 3 months from start of ADT (yes *vs*. no)**	**0.0050**	**0.462**	**0.270**	**0.792**	

ADT, androgen deprivation therapy; mCSPC, metastatic castration-sensitive prostate cancer; PSA, prostate specific antigen.

### Secondary Endpoints

**Figure 2:**
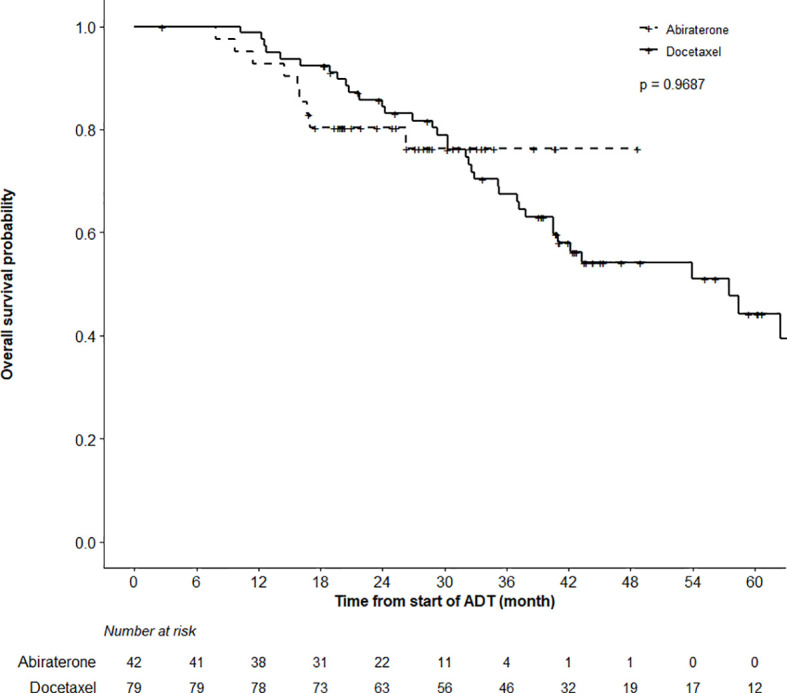
Overall Survival (OS) Analysis. Comparing OS in patients treated with abiraterone versus docetaxel using the log-rank test did not find a significant OS difference between groups (p = 0.9687). Patients alive or lost-to-follow-up were censored.

**Figure 3:**
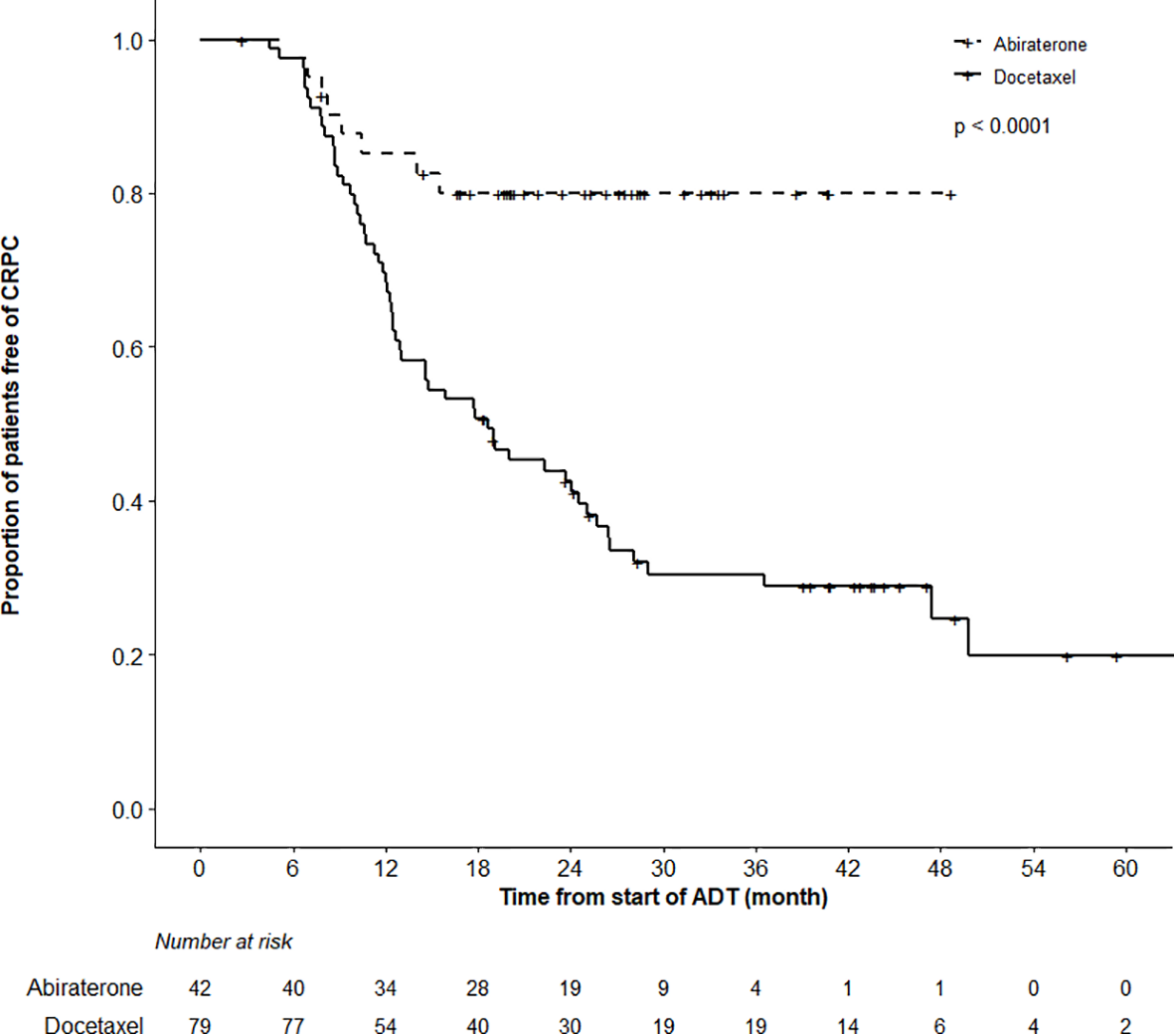
Time to Castration-Resistant Prostate Cancer (CRPC) Analysis. There was a highly significant difference in the time to CRPC between the abiraterone and docetaxel treatment groups (log-rank test, p < 0.0001).

While the actuarial median OS was not reached in the ABI cohort, it was 57.5 months (95%CI 40.5–62.5) in the DOC group (p = 0.97). OS at 12 months was 98.7% (95%CI 96.3–100%) and 92.7% (85.0–100%) in the DOC and ABI groups, respectively (**Figure 2**). In terms of time to CRPC, there was a statistically significant difference between the treatment cohorts (p < 0.0001); the actuarial median time to CRPC was 18.6 months (95%CI 12.6–25.1) in the DOC group and not reached in the ABI group (**Figure 3**). The actuarial median time to first line therapy for CRPC was 20.6 months (95%CI, 14.6–28.1) in the DOC group and not reached in patients who received ABI, statistically favoring ABI (p = 0.0086).

## Discussion

In our retrospective single-center cohort study, we found mCSPC patients treated with DOC to be younger, having less comorbidities, and experiencing shorter PFS compared to men undergoing ABI therapy. Similarly, the time to first line systemic therapy for CRPC was shorter in the DOC group. Since PFS and the diagnosis of CRPC were triggered by biochemical progression in the majority of patients, it is not unexpected that these outcome measures were favored by ABI over DOC, owing to the androgen synthesis inhibitory activity of ABI. However, there was no significant difference in OS between the two treatment modalities. As such our population-based results confirm the findings of the opportunistic comparison between the DOC and ABI arms of the STAMPEDE trial (arms C and G, respectively) (17).

Our study also documents the feasibility and effectiveness of both DOC and ABI under real-world conditions. In fact, 93.7% of our patients completed six cycles of DOC, and only approximately 5% of patients stopped either DOC or ABI because of adverse effects. With respect to DOC effectiveness, our DOC cohort was comparable to the CHAARTED population of men undergoing chemohormonal therapy regarding median age (65.76 *versus* 64 years) and rate of high volume disease (68.35 *versus* 66.2%), *de novo* metastatic presentation (65.82 *versus* 72.8%), visceral metastasis (20.25 *versus* 14.4%), and Gleason score 7 or higher disease (94.4 *versus* 94.1%) (24). The median OS was 57.6 months in CHAARTED and 57.5 months in our group of patients after a median follow-up of 39.6 months. Furthermore, the median time to CRPC was 18.6 (95%CI 12.6–25.1) and 20.2 (17.2–23.6) months in our cohort *versus* the experimental arm of CHAARTED, respectively (24). On the contrary, Lavoie et al. described inferior outcomes in their population-based series of 156 patients compared to our findings (33). However, their rate of high-volume disease was higher (79.5 *versus* 68.35%); fewer patients finished six cycles of DOC (81 *versus* 93.7%), and more men discontinued DOC because of toxicities (10 *versus* 5%).

With respect to ABI, our cohort was older compared to LATITUDE AND STAMPEDE-G, and situated between these two clinical trial populations in terms of risk factors such as LATITUDE high-risk and CHAARTED high-volume disease, *de novo* presentation, and rate of visceral metastasis. Although the relatively short median follow-up of 25.1 months of our patients precludes a definite comparison with LATITUDE and STAMPEDE-G, it is reassuring that 80.4% of our patients were alive at 2 years.

For the identification of predictive factors of clinical benefit, we focused on PFS as the primary endpoint in the entire study cohort. LATITUDE low-risk disease, achieving a PSA90 at 3 months, and older age at diagnosis of mCSPC were found to be independently associated with longer PFS. Interestingly, CHAARTED low-volume disease at baseline did not result in prolonged PFS in multivariable analysis. The LATITUDE risk definition might have better predictive potential because it incorporates not only tumor burden but also *de novo* presentation as factors of worse outcome (34). Deep PSA responses have been associated with superior outcome in men undergoing chemohormonal therapy or ABI combined with ADT for mCSPC (28, 30). Similarly, in our cohort PSA90 at 3 months was an independent predictor of superior PFS. With longer follow-up, we will be able to use our dataset for a predictive factor analysis using OS as the primary endpoint.

In the future, predictive clinical parameters such as the ones identified in our analysis might be used in conjunction with emerging molecular markers for therapeutic decisions. SPOP mutations appear to identify a group of mCPSC patients with excellent response to ADT alone (35). Conversely, Harshman et al. showed that elevated IL-8 levels predict shorter time to CRPC and OS independent of DOC administration, tumor burden, and recurrent *versus de novo* metastatic presentation, using baseline serum samples from CHAARTED (36). Luminal B subtype mCSPC was found to be associated with superior OS in men undergoing chemohormonal therapy compared to ADT alone, whereas men with basal subtype did not benefit from adding DOC (37). Combining ADT with apalutamide (and potentially other ARSIs) might be particularly needed in mCSPC with a high DECIPHER® genomic classifier score, low androgen receptor activity, and/or basal phenotype (38). The presence of germline DNA damage repair alterations predicts early progression from *de novo* mCSPC to mCRPC, yet such patients might be amenable to treatment with poly (ADP-ribose) polymerase inhibitors (39). Finally, deep tumor sequencing is expected to aid further in the precise molecular classification of mCSPC for therapeutic decision making (40–42).

Some limitations regarding our analyses are worth mentioning, including the retrospective data collection and limited sample size. Furthermore, the single institution nature of our study could affect the external validation of our findings, as could differences between the treatment cohorts in terms of age, comorbidities and time of follow-up among others. Regarding the latter, the relatively short median follow-up in the ABI arm is explained by the only recent availability in Canada of ABI for mCSPC. Furthermore, imaging was timed according to treating physicians’ discretion, which could impact the assessment of PFS.

To sum up, the findings presented herein support the feasibility and effectiveness of combining DOC or ABI with ADT for men with mCSPC under real-world conditions. The presence of high-risk mCSPC as per LATITUDE criteria, lack of a PSA90 at 3 months, and younger age at diagnosis of mCPSC predict shorter PFS and as such may identify a cohort of patients in need for further refinement of the initial management.

## Data Availability Statement

The raw, de-identification data supporting the conclusions of this article will be made available by the authors without undue reservation.

## Ethics Statement

The studies involving human participants were reviewed and approved by the Research Ethics Board of Sunnybrook Research Institute. Written informed consent for participation was not required for this study in accordance with the national legislation and the institutional requirements.

## Author Contributions

JB and UE conceived and designed the study. JB, MK, and AS collected and assembled the data. LZ performed the statistical analysis. JB, MS, and UE analyzed and interpreted the data. JB and UE wrote the manuscript. All authors contributed to the article and approved the submitted version.

## Funding

This study was made possible by support from the Joseph and Silvana Melara Cancer Research Fund (Toronto, ON, Canada) to UE.

## Conflict of Interest

UE has received honoraria from Janssen Inc. Canada for consulting and educational activities. 

The remaining authors declare that the research was conducted in the absence of any commercial or financial relationships that could be construed as a potential conflict of interest.
